# Development of a childhood food and nutrition security observatory: experience of an interinstitutional initiative in the state of São Paulo

**DOI:** 10.1016/j.jped.2026.101547

**Published:** 2026-04-25

**Authors:** Karina Viani, Marcos Sakurada, Aracelia Costa, Mauro Fisberg, Priscila Maximino, Raul Cutait, Committee of Experts – Childhood FNS Observatory, Adriana Alvarenga, Vanessa Tamara Ferreira, Dirce Maria Lobo Marchioni, Luciana Phebo, Vanuzia Teixeira, Aline Yukimitsu

**Affiliations:** g Fundo das Nações Unidas para a Infância (UNICEF), Brasília, DF, Brazil; h SindHosp, Federação dos Hospitais, Clínicas e Laboratórios do Estado de São Paulo (Fesaúde), São Paulo, SP, Brazil; i Departamento de Nutrição, Faculdade de Saúde Pública, Universidade de São Paulo, São Paulo, SP, Brazil; j Federation of Industries of the State of São Paulo (FIESP), São Paulo, SP, Brazil; a Universidade de São Paulo (USP), Faculdade de Medicina, Departamento de Pediatria, São Paulo, SP, Brazil; b Federação das Indústrias do Estado de São Paulo (FIESP), São Paulo, SP, Brazil; c Instituto de Pesquisa PENSI, Centro de Excelência em Dificuldades Alimentares, São Paulo, SP, Brazil; d Universidade Federal de São Paulo (UNIFESP), Departamento de Pediatria, São Paulo, SP, Brazil; e Hospital Sírio-Libanês, Departmento de Cirurgia, São Paulo, SP, Brazil; f Universidade de São Paulo (USP), Faculdade de Medicina, Departmento de Cirurgia, São Paulo, SP, Brazil

**Keywords:** Dashboard systems, Food security, Diet, food, and nutrition, Child health

## Abstract

**Objective:**

To describe the development of the FIESP Observatory for Childhood Food and Nutrition Security (FNS), a digital platform designed to organize and integrate public data on children aged 0 to 10 years in the state of São Paulo, Brazil.

**Methods:**

This descriptive study involved three main stages: (1) a literature review and mapping of national and international observatories on health, nutrition, and childhood to identify best practices and gaps; (2) establishment of an expert committee of professionals in nutrition, public health, and public policies to guide indicator selection and ensure technical quality; and (3) technical development of the platform, including automated data extraction, processing, and integration into thematic dashboards. Public databases from official sources were incorporated, structured across six thematic axes: demographic, socioeconomic, FNS policies, nutritional, health, and educational profiles.

**Results:**

The observatory was launched in May 2025 and made freely available online. It features six interactive panels and a “Your City” dashboard that consolidates key indicators for each municipality in São Paulo. Since its launch, it has received >9600 visits, with 4755 active users. The tool enables segmented analyses by geography, time, and demographics, providing evidence to support policy design and monitoring.

**Conclusions:**

The Observatory represents an innovative and scalable model for consolidating childhood FNS data. By offering free, intuitive, and continuously updated access to official indicators, it supports policymakers, researchers, and civil society in addressing childhood food insecurity and advancing the right to adequate and healthy food.

## Introduction

Food and Nutrition Security (FNS) in childhood is a fundamental human right and an indispensable condition to guarantee the healthy growth and the physical, intellectual, and emotional development of children. The lack of adequate nutrition in the first years of life is associated with an increased risk of morbidity, delayed development and compromised human potential, directly impacting public health and the future of society [1,2]. In the state of São Paulo, it is estimated that 9.2 million people, 20% of its population, face food insecurity, with 6.7 million experiencing mild, 1.5 million moderate and 1 million severe food insecurity [3]. Among Brazilian households with children, 30 to 50% face severe or moderate food insecurity [4]. Furthermore, childhood obesity is a growing concern: 22% of Brazilian children aged 0 to 10 years are overweight or obese [5]. Despite the topic's relevance, obtaining systematic information on childhood FNS remains a challenge in Brazil. Available data in national public databases is scattered across different platforms, with heterogeneous formats and difficult to access, limiting its use by public administrators, health professionals, and researchers.

The “Feed the Future Program” (in Portuguese “Programa Alimentar o Futuro”), developed by the Superior Council for Social Responsibility (Consocial) of the Federation of Industries of the State of São Paulo (FIESP), in partnership with the Center for Industries of the State of São Paulo (CIESP) and the Industry Social Service of São Paulo (SESI-SP), has the mission of promoting the FNS of children aged 0 to 10 years in the state of São Paulo [6]. One of the Program's strategies was the development of the FIESP Observatory for Childhood Food and Nutrition Security, designed as a digital platform that organizes, integrates, and makes available relevant public data for the improvement and monitoring of public and private policies related to FNS for children in the state of São Paulo. This is an inter-institutional initiative led by FIESP in partnership with the United Nations Children's Fund (UNICEF), the PENSI Institute – São Paulo, Brazil, and the Federation of Hospitals, Clinics and Laboratories of the State of São Paulo (Fesaúde).

The initiative seeks to improve public and private policies, support the continuous monitoring of FNS across the 645 municipalities of São Paulo, and provide managers, researchers, professionals, and the public with a free and accessible source of information. Ultimately, it aims to strengthen the right to adequate and healthy food in childhood. The objective of this study is to describe the development of the FIESP Observatory for Childhood FNS, designed as a tool for aggregating public data on children aged 0 to 10 years in the state of São Paulo, Brazil.

## Methods

The development of the observatory was informed by a narrative, non-systematic review of the literature on childhood FNS, as well as an exploratory search for similar national and international online observatories in the fields of health, nutrition, and childhood. This exploratory survey aimed to identify structural features, thematic scope, and best practices to inform the observatory’s design. Strategic partnerships were established with national and international reference institutions and organizations with a strong record in promoting the right to healthy and adequate food in childhood, namely Unicef Brazil, Instituto PENSI – São Paulo, and Fesaúde. These partnerships were designed to reinforce the observatory’s governance and broaden its scientific and institutional bases.

To ensure technical quality and content relevance, a Committee of Experts was established, composed of eleven members with expertise in nutrition, pediatrics, public health, public policy, and data analysis. The committee included the project coordinator (a nutritionist), public policy specialists with extensive experience in government, academic researchers, a data analyst, representatives from UNICEF, healthcare sector representatives (Fesaúde), and members of Instituto PENSI. The Committee of Experts members served on a voluntary basis, providing continuous feedback throughout the planning, development, and validation stages of the observatory, through both online and in-person meetings.

As the study did not involve human subjects, identifiable data, or biological material, formal approval by a Research Ethics Committee was not required. In terms of technical infrastructure, the observatory was developed to enable:

• Automatic data updates, aligned with the frequency of official public sources.
• Easy navigation through interactive menus that allow switching between different thematic research areas.
• Dynamic filter functionality for segmented viewing by geographic region, age group, gender, etc.
• "Management at a glance" dashboard model for a specific city or region.

The observatory was structured around six thematic axes: demographic profile, socioeconomic profile, FNS policies, nutritional profile, health profile, and educational profile. For each axis, the Committee of Experts identified public data sources, and the indicators for inclusion in each axis were defined based on their relevance and critical analysis of their quality and usefulness in this context. The platform also includes a "Your City" dashboard, which presents a summary of the main indicators for each thematic axis, bringing together, on a single screen, essential information about the selected city or region. This function aims to facilitate quick and integrated access to relevant data across different areas of the state of São Paulo.

The procedures for extracting, processing, and storing the data used in each of the thematic dashboards are described below. Supplemental Table 1 provides further details on the descriptive data and transformations applied to the data sources.

### Demographic profile

Demographic data were extracted from the 2022 census of the Brazilian Institute of Geography and Statistics (IBGE) [7]. The tables were manually retrieved via the IBGE Automatic Recovery System (SIDRA) (Tables 9514 and 9606) in .xlsx and .csv formats. Data processing included aggregations and transformations using the *Power Query* tool, particularly for corrections in race/skin color categories with missing data ("not reported"). The resulting file was stored in .csv format.

### Socioeconomic profile

Data from Cadastro Único (CadÚnico), the Bolsa Família Program, the Annual Report of Social Information (Rais), and the Human Development Index (HDI) were included [8., 9., 10.]. The databases were extracted in .csv, .txt, and .parquet formats. Automated scripts in the *Azure Data Factory* (ADF) service using *Data Flows* enabled data processing, merging, and standardization, as well as the creation of derived indicators, such as families experiencing poverty and low income. The average salary was obtained by dividing the total salary by the number of active employment contracts, calculated using *Microsoft Power Business Intelligence* (*Power BI*).

### Educational profile

Microdata from the school census, by the National Institute of Studies and Educational Research Anísio Teixeira, were used (INEP) (2021–2024) [11], manually extracted in .zip format containing .csv files. The data were processed with ADF, standardized in .parquet format and validated with the official INEP panels.

### Nutritional profile

Two main sources were incorporated: Food and Nutrition Surveillance System (SISVAN) (for indicators of nutritional status and food consumption)[5] and Brazilian Food Insecurity Scale (EBIA) [3]. SISVAN was accessed through manual and automated extractions using *Python* scripts, which enabled the systematic collection of anthropometric and food consumption data. The processing steps involved transforming formats (.xls, .csv) into .parquet using ADF. EBIA was accessed through *Power BI* dashboards from the Ministry of Social Development and Assistance, Family and Fight against Hunger (MDS), with adjustments made in *Power Query*, such as standardizing decimals and reference years.

### Health profile

Child mortality data were obtained from the Mortality Information System (SIM), via the OpenDataSUS platform, extracted in .csv format and processed in the *Pandas* library and in ADF [12]. The transformations included age conversion, creation of time variables (year, month), and filtering for the state of São Paulo. Additionally, data from the National Immunization Program (PNI) were used [13], as well as from the Secretariat of Primary Health Care (SAPS) [14], manually extracted in .xlsx and processed with *Power Query*.

### FNS policies

The National Food and Nutrition Security System (SISAN), created by Federal Law No 11,346 of September 15, 2006, is a public management structure that coordinates actions from different sectors and spheres of government, with the participation of civil society, to guarantee the human right to adequate food. At the federal, state, and municipal levels, the Interministerial Chamber for Food and Nutrition Security (CAISAN) coordinates intersectoral policies focused on FNS. In the cities, the Municipal Food and Nutrition Security Councils (COMSEA) play a fundamental role in oversight and social participation, promoting local mobilization and serving as forums for dialogue between government and civil society to strengthen public food and nutrition policies [15].

Information on cities’ adherence to SISAN, the existence and activity of CAISAN and COMSEA, and the acquisition of food from family farming for the National School Feeding Program (PNAE) was obtained via official documents and *Power BI* dashboards [15,16]. The extraction included *Python* scripts for *webscraping* and reading .pdf, .html, and .xlsx files. The processing was mostly done in ADF, and the final files were stored as .parquet or .csv.

### Storage, integration and validation

All data was stored in the *Azure DataLake Storage Gen2* data storage service, ensuring centralization, version control, and security. The tables were integrated into interactive dashboards in *Power BI*, with cross-validation between the original sources and the processed data.

## Results

The exploratory search identified national and international online observatories in the fields of food and nutrition security, public health surveillance, and child health. These initiatives varied in scope, ranging from data dashboards and policy monitoring platforms to research dissemination hubs. Common features included the use of publicly available secondary data, thematic dashboards, and periodic updates [17., 18., 19., 20., 21., 22., 23.]. These elements informed the structural and thematic organization of the present observatory.

The FIESP Observatory for Childhood FNS was publicly launched on May 29, 2025, and is available on the Feed the Future Program website (URL: https://alimentarfuturo.fiesp.com.br/observatorio-painel.html). The platform has been widely publicized through institutional networks, the press, newsletters, and internal communication channels. Since its launch, the observatory has received >9600 visits, with 4755 active users, indicating interest in the tool.

**Figure 1:**
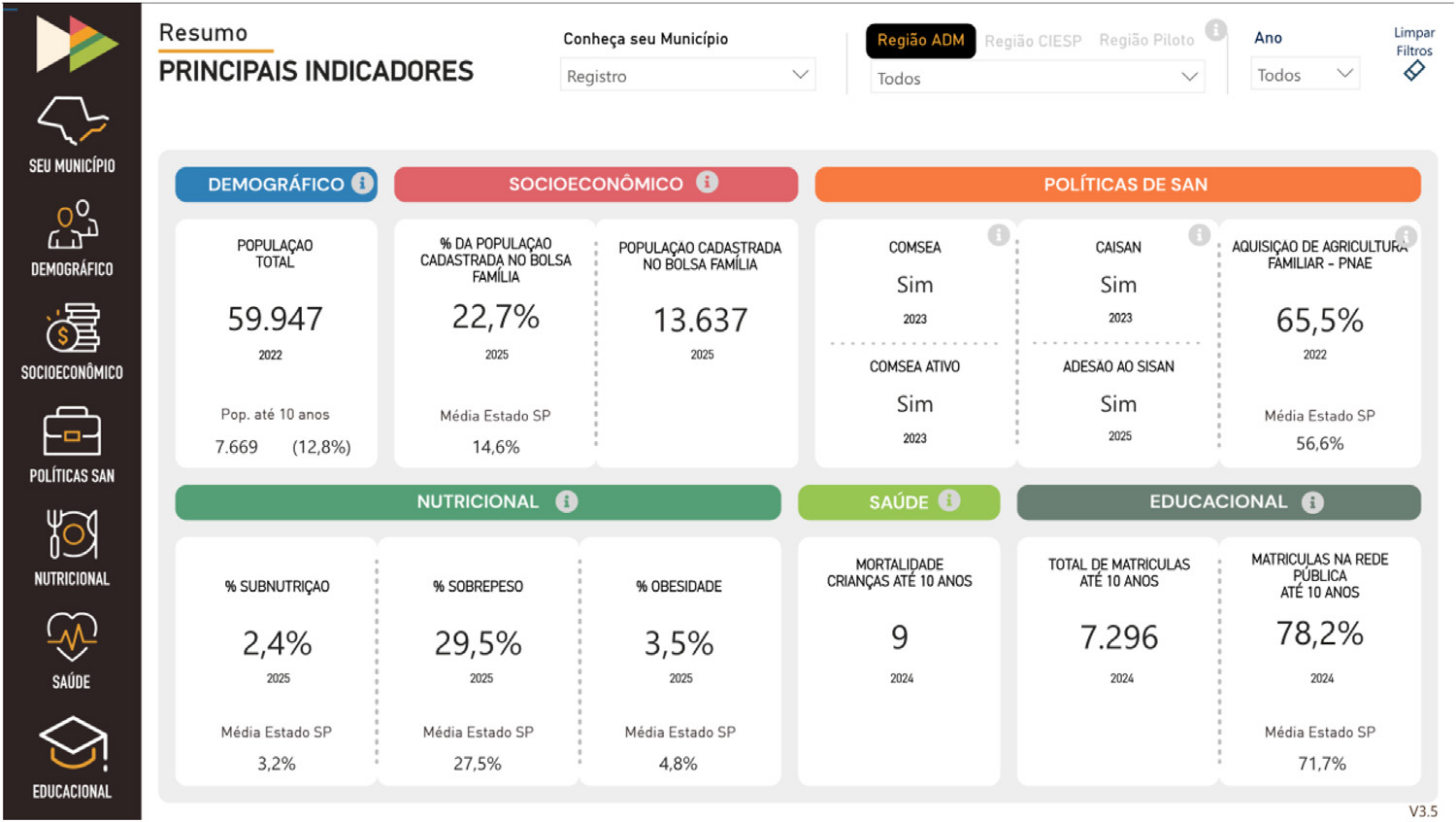
Print screen of “Your City” dashboard of the FIESP Observatory for Childhood Food and Nutrition Security.

Source: https://alimentarfuturo.fiesp.com.br/observatorio-painel.ht ml.

**Fig. 2:**
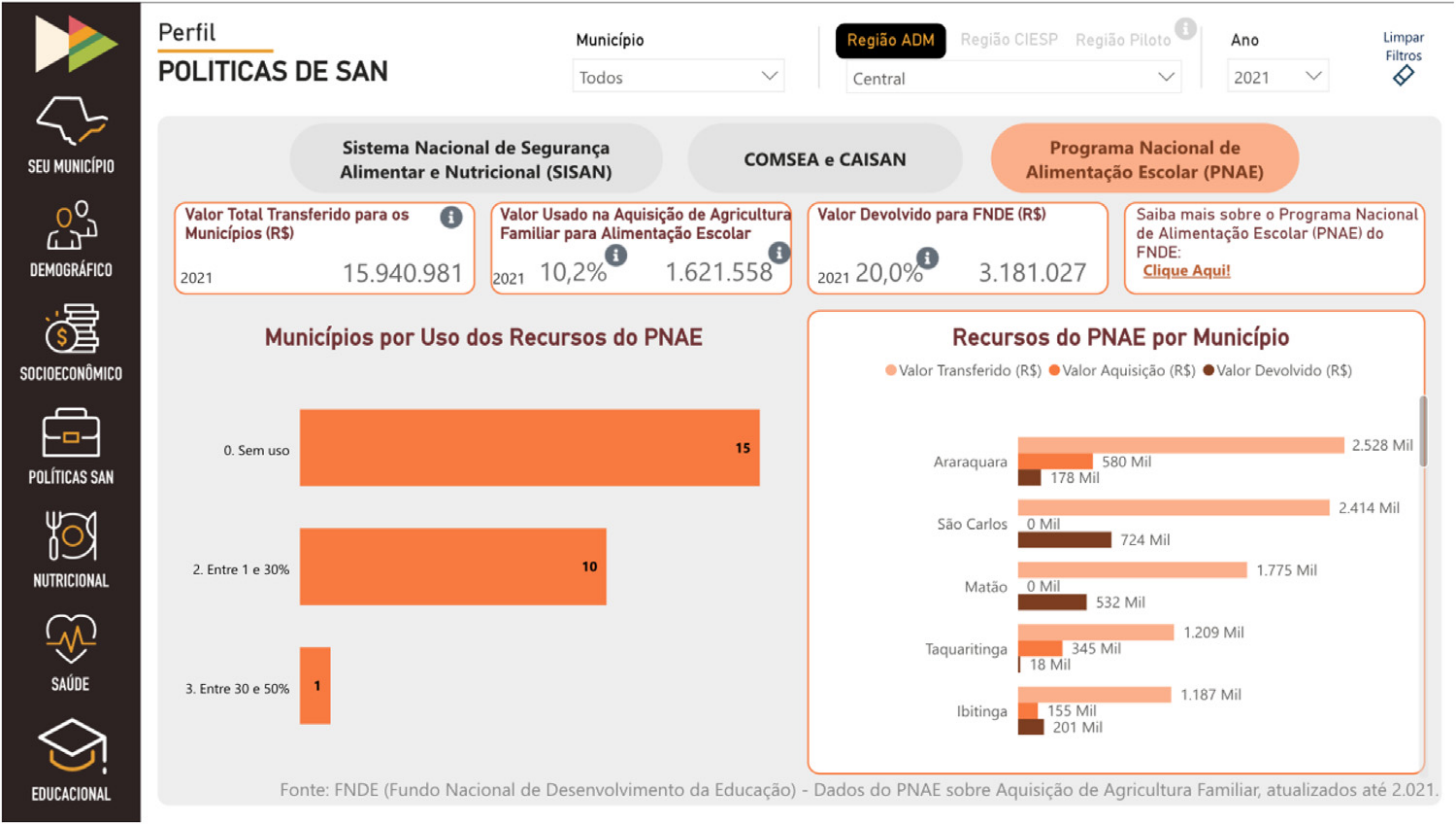
Print screen of “FNS policies” dashboard of the FIESP observatory for childhood food and nutrition security.

Source: https://alimentarfuturo.fiesp.com.br/observatorio-painel.html. FNS, Food and Nutrition Security.

The platform consists of six interactive panels related to each thematic axis (demographic profile, socioeconomic profile, FNS policies, nutritional profile, health profile, and educational profile), in addition to the "Your City" dashboard, illustrated in Figure 1, which presents a summary of the main indicators for each thematic axis. Each panel gathers indicators from official and public sources, such as IBGE, SISVAN, and SIM, presented visually in numbers, percentages, tables, maps and graphs. For example, the nutritional profile panel includes data on overweight, stunting, and breastfeeding; while the public policy panel, illustrated in Figure 2, allows viewing the percentage of resources allocated to the purchase of food from family farms within the scope of PNAE. The dashboard structure was developed to support usability across different user profiles, with geographic, temporal, and demographic filters, enabling local and regional analyses.

Automatic data updates are implemented according to the release schedule of each official public data source. As the observatory relies exclusively on secondary data from public databases, the update frequency varies by indicator, ranging from monthly and annual releases to census-based periodic updates. The system is programmed to incorporate newly released data automatically once it becomes publicly available.

## Discussion

This study describes the development of the FIESP Observatory for Childhood FNS as a public data aggregator, resulting in an accessible instrument to support public administrators, health professionals, researchers, and civil society in analyzing the food and nutrition situation of children aged 0 to 10 years in the state of São Paulo. By organizing relevant indicators into easy-to-navigate thematic dashboards with automated updates, the observatory helps fill a gap in the systematization and transparency of information on FNS in children. In addition to supporting the management of local public policies by offering concrete and unified data that guide the goals to be achieved, the observatory also responds to specific demands from public administrators, providing essential evidence for the development and implementation of policies to combat food insecurity.

Compared to other national and international health and nutrition monitoring initiatives, this observatory presents some important innovations. While most public health observatories in Brazil focus on specific areas, such as mortality, school meals, or chronic conditions, this tool aims to integrate multiple dimensions of FNS into a single platform, with an exclusive focus on childhood. The modular and thematic structure of the panels, the territorial basis by city and region of the state, the possibility of different filters, and the intuitive interface are features that expand its applicability and accessibility, even for users without specialized technical training.

One of the observatory's main challenges concerns the quality, completeness, and coverage of SISVAN data, which are essential for guiding analyses of children's FNS. SISVAN data depend exclusively on information recorded in the e-SUS Primary Health Care system and therefore reflect only the population accessing public primary care services and having anthropometric measurements and food consumption markers registered. Consequently, the system does not represent the total population of children aged 0 to 10 years. In addition, coverage is heterogeneous across states and municipalities, and the completion rate of food consumption markers is low in many localities, which may compromise the representativeness and territorial comparability of dietary indicators. Previous studies have identified limitations such as low monitoring frequency, lack of data on key variables (such as race/skin color), and inconsistencies resulting from the integration of different sources that can affect SISVAN’s analytical quality [24., 25., 26., 27.]. To mitigate these limitations, the authors incorporated the estimated population coverage percentage in the observatory, promoting transparency and a more comprehensive view, allowing users to contextualize the results. Nevertheless, findings derived from SISVAN data should be interpreted with caution, particularly in local analyses.

The strengths of this initiative include its originality, free and open access, comprehensive population coverage, intuitive interface, and automated updating. Its inter-institutional nature, combined with oversight by a Committee of Experts, enriches the observatory by ensuring technical rigor in the selection of indicators, reinforcing its scientific credibility. Nonetheless, certain limitations must be acknowledged, particularly those related to the underlying databases, which constrain the inclusion of additional filters and, consequently, the possibility of more granular analyses. Furthermore, the geographic scope is restricted to the state of São Paulo, as determined by its connection to the Feed the Future Program, which imposes an inherent regional limitation. Despite these constraints, the observatory’s methodological and technical model demonstrates strong scalability potential and may serve as a reference for similar initiatives in other regions of Brazil and internationally. Beyond its technical innovation, by systematizing and publicly disseminating regional data, the observatory provides an evidence base capable of informing and strengthening the formulation, monitoring, and evaluation of regional public policies in childhood FNS.

Next steps include periodically updating the platform’s data in line with new updates from official databases, as well as including additional indicators and filters defined in collaboration with the Committee of Experts during quarterly meetings. The implementation of customized reports by city or region, automatically generated from combinations of indicators and filters, is also under consideration to enhance the tool’s practical utility for users. In parallel, efforts will focus on expanding the observatory’s reach through technical events, scientific societies, academic institutions, and institutional networks. The adopted model demonstrates the feasibility of replication in other regions of the country, with the potential to strengthen FNS surveillance in Brazil and support the formulation and monitoring of evidence-based public policies. Furthermore, international experiences, such as the Global Nutrition Report [28] and the World Health Organization's (WHO) Global Health Observatory, [20] demonstrate the relevance of monitoring platforms that integrate data and promote the use of evidence to inform public policies. The inclusion of this international perspective expands the possibilities for dialogue and cooperation between institutions, reinforcing the observatory's credibility and applicability in diverse contexts.

The development of the FIESP Observatory for Childhood FNS represents a significant advancement in monitoring the FNS conditions of children aged 0 to 10 years in the state of São Paulo, Brazil, with potential implications for the entire country. Consolidating public data in an accessible and integrated platform strengthens the evidence base for planning more effective public policies, fosters intersectoral actions grounded in scientific evidence, and supports the work of public administrators, researchers, and civil society. Through continuous updates, the observatory seeks to remain a timely and reliable tool for decision-making, thereby contributing to the fulfillment of children’s right to adequate and healthy food.

## Funding

Federação das Indústrias do Estado de São Paulo (FIESP).

## Data availability

The data that support the findings of this study are available from the corresponding author.

## Conflicts of interest

Karina Viani: The author acts as a technical consultant for the "Alimentar o Futuro" social program of the Federation of Industries of the State of São Paulo (FIESP), focused on promoting food and nutrition security initiatives. This position is independent of the food industry and did not influence the development or content of this manuscript.

Marcos Sakurada, Aracelia Costa: The authors work for the "Alimentar o Futuro" social program of the Federation of Industries of the State of São Paulo (FIESP), focused on promoting food and nutrition security initiatives. These positions are independent of the food industry and did not influence the development or content of this manuscript.

Mauro Fisberg: The author is a counselor of the Superior Council for Social Responsibility (Consocial) of the Federation of Industries of the State of São Paulo (FIESP). This position is independent of the food industry and did not influence the development or content of this manuscript. MF is a member of scientific Boards (SBAN, SPSP) and is a speaker for food, pharmacy and health organizations. These jobs did not interfere with the development of this manuscript.

Raul Cutait: The author is the president of the Superior Council for Social Responsibility (Consocial) of the Federation of Industries of the State of São Paulo (FIESP). This position is independent of the food industry and did not influence the development or content of this manuscript.

## Appendix Supplementary materials

Image, application 1

## Footnotes

Supplementary material associated with this article can be found in the online version at doi:10.1016/j.jped.2026.101547.
