# Supplementary material for: Development of a childhood food and nutrition security observatory: experience of an interinstitutional initiative in the state of São Paulo
Source: J Pediatr (Rio J). 2026 Apr 25;102(3):101547. doi: 10.1016/j.jped.2026.101547 (PMC13133930; doi:10.1016/j.jped.2026.101547)
Supplement: Supplementary file 1 [file mmc1.docx]

**JPED-D-25-00468_Supplementary Material**

**Supplemental Table 1** Description and transformations applied to the data sources of the FIESP Observatory for Childhood FNS.

| Source Description | Responsible Institution | Theme^a^ | Link URL Source | Period^b^ | Extraction | Transformation | Description |
| --- | --- | --- | --- | --- | --- | --- | --- |
| Demographic Census | IBGE | 1 | [Link](https://sidra.ibge.gov.br/tabela/9606) | 2022 | Manual download | Correction Population by race/color | Insert rows for the "Color or Race" column with "Not Provided" for the population that does not fit the available options. This way, the table is equivalent to the total population of São Paulo. |
| HDI | UN | 2 | [Link](https://www.undp.org/pt/brazil/idhm-municipios-2010) | 2010 | Manual download | Municipal Standardization | Add the column "UF" from "Municipality"; format "Ranking IDHM 2010" to number type; standardize "Municipality" in the cases: "Embu" to "Embu das Artes" and "Embu das Artes-Guaçu" to "Embu-Guaçu"; create the column "IDHM Range" with: 0 - 0.699 (Low and Medium), 0.7 - 0.799 (High) and 0.8 or more (Very High). |
| Salaries | RAIS | 2 | [Link](https://www.gov.br/trabalho-e-emprego/pt-br/assuntos/estatisticas-trabalho/microdados-rais-e-caged) | 2021-2024 | Manual download | Aggregation by year, active links, average salary calculation | Convert .txt to .parquet; create the "Year" column, merge the files by year; filter only active contracts "EMP AS OF 12/31" = 1; format the monthly and average compensation columns to the Brazilian standard ("," por "."); aggregate by sum of compensation values ​​and count of active contracts to calculate the aggregate average.  Note – premise: The average salary is calculated by the ratio of the sum of the Salary Range Dezem Nom by the count of Active Contracts as of 12/31, considering the premise "Active Contracts as of 12/31 equal to 1." |
| Registered on CadÚnico | SAGICAD | 2 | [Families](https://aplicacoes.cidadania.gov.br/vis/data3/v.php?q%5b%5d=oNOclsLerpibuKep3bWChLNe09Gv17lljax%2FYWyAYmqqdH9%2BaWKEkWaXbWTZ6ayanbWUndqdiLSYmcrGbtCen9DgiG%2BiqaGt3nSIwayaes%2BS0J6gvKuEZJurlp60n666qpKSx5TWsJiYtrOVqLuadbSswrtam7bHlNecY5SrrGVweJSd2p2ItJiZysZu0J6f0OCIb6Kpoa3edIjBrJp6z5LQnqC8rIFkm6uWnrSfrrqqkpLHlNawmJi2s5Wou5p1tKzCu1qfvdGWyW5njdp%2Fa26Dm5vlrLKJnY7D1JileJm%2B58CZd4Oor%2BZcu62djsTAZptuksDcsW%2BiqaGt3nSzr6OgvJxu0J6f0OCIb6%2B9ol30WrC9mJm81JbPZXPL2rOVqaeYm91lfXdXWnfEosupmNDeslx8tpSg2qasgWhetsSUzmljhpzKb6Kpoa3edLOvo6C8nG7Qnp%2FQ4Ihvr72itsqurryrlrvCl89dp8zvrqBcrJpa35q6EeSZwMKmiqah0N6%2FnbCpqFrnqG2RmJG41KfcrFMgFbudn7dYi%2B6au8KgkbjFmIqhmH3hrqH%2F9aGj2qxts6RNysqn3572BD7wo1ysmlrescHAnJq4gaPZn6XC9a5Upbaoneuiwa%2BqTcXQU62el77uwaaraPjU56KwvVp%2BzMKh3qaXvt%2ByVKCtVaDaphD7o5a41FPPqlPQ5MGpnQvc%2FRyobbKcTcfQldyirb6btqKvq6ej7ZrAbqWcd6SUzp6m0e28VP%2Fio6PcqHCfrI7F1ZzOnpfCm7GZXK6WpzzmubeYoHfGoIqwnNHwrvfjC9ipmZ2ybqecudOY5J5ffe6ym7G2mamZmm20mJbPwlPOrFOt7bybrqmim5l7vLqqjnenlNcA4Mnkrl5oaJ6o7Jy%2Ft6uOyoGh2V12vt%2Bup7C6pFo807u3mpx6sqjLq6fG366YoWiZn5mfrrv62sPKlN1dl8Kbr5WlwJZa6567sphXgYGc2LCWz%2BTBla9oo6mZfK6ymKDL06KKAM3L5LCjX5mqm%2BettrKYkbyBl89dmb7oEOGosZatmZy8u1efvM%2BXy12jwu1tl524nq7aWbqzpaC4zVPLsfYGm7qZpbdVrdqlEO%2BplsaOoC3qocbovFRkmKSc657Hr1dYd6OU07WUfe2yoqCpXlrip8CxqZbLwqaKq6J9vq6YnbuprOhZEOillrrQVruylMvvtpidrJpa3Z5ttJiaGg6f056mfd68oVy6mqjdmm2%2BnJ93xJTapqe%2Bm7qZqruWppmasLekjnfFmIqqmMbqbaedtPjb66K8e6TwBM%2Bc16xdh6Vtnaq7mKzira7BV5vGgXbLoZTQ77%2BjXAvPqOKcvMqnaNPdr5xtZZCofWdpeGaOqWmHfmdnh5GNpQ%3D%3D&ag=m),  [People](https://aplicacoes.cidadania.gov.br/vis/data3/v.php?q%5b%5d=oNOclsLerpibuKep3bWDhLNe09Gv17lljax%2FYWyAYmqqdH9%2BaWKEkWaXbWTZ6aykobuomdyasYmdjsPUmKWjlMnusm93rpam7J6IiaqixIShya2Y0O6sZHN4lJ3anYi0mJnKxm7Qnp%2FQ4Ihvoqmhrd50iMGsmnrPktqiptDafWtsp2ZuqZiwr5tovcKf3aJuw9y5p6GDcKDapcCzcmjK1qCNq5LN4MCnm3lpaticrrJyk7jNps94mb7nwJl3g5ub5ayyiXKgzM5W5V6WzNy5ma%2BrmmK5p6y%2BnKDKwGabbpLA3LFgbHFiYtyorrqcoLrGW6qrks3gwKebeGxq2JyusmNdgIyW2Z6fwu6wmWSIo5npnsDBll2OkZKbcWO83q6YaHheY5q2iLSYmcrGbtCen9DgiG%2BiqaGt3nSIwayaes%2BS2qKm0NqAZW2nmJvddLOvo6C8nJnLqabCtoianbSon7R0wMOkUNKCltmen8LusJlkiKOZ6Z7AwZaQuMVfmmZgwOquoKG7mJ%2Bhebutp5LK1JKdbmS83q6YaHheW%2FZ0s6%2BjoLycmcuppsK2iJqdtKiftHTAw6SpqNaU2LGcwdyxmVy8pK7apW2ynE3HxqbdrJTQm7air6uno%2B2awG6lnHeklM6eptHtvFT%2F4qOj3Khwn6yOxdWczp6XwpuxmVy4mq3sqK7BV5bF1Jbcpqe%2B7m2iq2h4m92awMKpnHckzdimlsybsqFcrpanPOa5t5igd8agirCc0fCu9%2BML2KmZnbJunKXL05jXnlPN6q%2BmocKWXcqurryrlrvCl89dl8KbvZmvu6Sb7Fm2vKqQycqny7BTy%2Bptd52slq3tq7xu%2BsfFypbZXZjKm7OVqQvipuKawG6cmnfUnN6ylCAiENeraJmfmam8sKmS0cJWu7KUy%2B%2B2mJ2smlrdnm2%2BnKDK0JTdXZzL7rCmpbyWrZmnvG56jrvCpt6von0%2B56Klq6Ra3qZttJiaGg6f056mfeC6VK%2Bxqa%2Fa%2FPQR2px3xZiKraK%2F7bKunXRVrd6gwrybnHfCU9CenNXcbZiraIWs6KC%2Fr6SOd6Oi1rCUfcGuof%2F1oaPaY3CfrI7F1ZzOnpfCm7GZXLiareyorsFXlsXUltymp77ubaKraHib3ZrAwqmcdyTN2KaWzJuyoVyulqc85rm3mKB3xZiKn5TG865Urq2jntpjd3GIorjPp9OhlMHgbZihaKWf7Ky8r6pNwM%2Bmza%2Bc0dzAVKq3VX3ana7Bq5%2FGgfYEq5zA6m2ZqWibm%2Bb8%2BrqgjsqBltmqU8%2Fgu5idaKWf61mwr6eWy8JT16Kh0Ny5VJ28%2BOOZprK3pk3Kwp8t3qXG6m2h%2F%2FWjo%2BaobXaHnLnTmOSeU4ibj5WlwJZa6567sphWerKoy6unxt%2BumKFomZ%2BZqbLBqpy41FPTq6bA7baonbtVqOhZkK%2BbjsrVpdld9vfptperaJqnmZ%2Buu%2Fraw8qU3V2WzOhtpqG2mZuZqbLAV5C40ZzenlPK4LunnbRVm9yiuq9XkbyBoM%2Bmon3urqD%2F6aej6Fm6EeSbwM6ilGdd2euIsLjEZ2qrbHp%2BalqHkoeabW2Nq4dkbKJw&ag=m) | 03/2023-03/2025 | Download via HTTP | Aggregated families and people | Join the Registered People and Registered Families tables in CadÚnico; create the INSCRITAS_BAIXA_RENDA and INSCRITAS_TOTAL columns for Persons and Families; fill nulls with zero. |
| Bolsa Família beneficiaries | SAGICAD | 2 | [Families](https://aplicacoes.cidadania.gov.br/vis/data3/v.php?q%5b%5d=oNOclsLerpibuKep3bWFhLNe09Gv17lljayEYWx8YmqqdH9%2BaWKEkWWXbWTZ7MGYoaelnN%2BYfa2dpbbTmdqgko62s5Wou5p17avCs3JovcKf3aJumO7CoV%2B5qZ7emL2wnYyHwJninKXD67CTboObm%2BWssomrn8zGbqWjlMnusm93u6qnnKrBspyMx8OZyW2Sw%2FOspqK4mJmsdLOvo6C8nKfcspiYtrOVqLuadbSswrtansvFmMmtlcPafZOiwJSs36mwrWtovcKf3aJu0e3CmXeDm5vlrLKJcqDMzlbbsZfC2r2Woqdmmd%2BxrMCdnbrAZKWjlMnusm%2BwuqqftHSzr6OgvJxu3bKggOzBmKGnpZzfmH6tnaW205naoJKPtrOVqLuade2rwrNyaL3Cn92ibpjuwqFfw1ad6Jq5s6qQvIlz27GXwtq9lqKnZpnfsazAnZ26wGSWbVyI3ryVqK2ond5hjb%2BrkbzAo8yjko7as6ybupuq3Jh%2FemdWeN5u0J6f0OCImp20qJ%2B0dLOvo6C8nG7dsqCA7MGYoaelnN%2BYfq2dpbbTmdqgkpC2s5Wou5p17avCs3JovcKf3aJumO7CoV%2B5qZ7emL2wnYyIwJninKXD67CTcIObm%2BWssomrn8zGbqWjlMnusm93u6qn9YrCr6WhwMWUzqJTweBtmp21%2BOfloq7BV5LEgabTsai%2BPvT337dVnt5Zssarn7zOlIqtor%2Ftsq6daJ6o7Jy%2Ft6uOyoGh2V12vt%2Bup7C6pFo807u3mpx3r%2FbtjFO%2F4LuZorGYozzav7eYoHfFooqNpcziv5WpqVV86KXAr1dzuM72F6mcvp6eqZ22qaPdmrGzV5G8gZnLqvYK57aVr2iap5mstsKsjhoI9g2sU8HgbaSrqqef85ptt6WgutOc3p6mfem8VH%2BpmZvsrb%2B9V%2FDxz5zNrFOrPtCDXKqaqN6ftrGg8PjTnMuwU8HqbYSut5ys2qaubnmcw9SUioOUyj76oKWpWIvumrvCoJG4xZiKoZh94a6h%2F%2FWho9qsbbKcTbnCnOKeU8%2Fgu5idcl9a4qfAsamWy8Kmiquifb6umJ27qazoWRDopZa60FO4ALasm6%2BZqq2bo9yiEO%2BplrjUU86sU63tvJuuqaKbmXu8uqqOd6eU1wDgyeSuV429lqjtorGvm5J3xZiKo5TKPvqgpamoWtyoum6pksXFlIqtmM%2BbsJWssambmaayvKqOw4GUzaagvpuxmVy1mqPoWcCvo%2FD405zZaqAgKLudqbdfZKNZtryqkMnKp8uwU8vqbXedrJat7au8bvrHxcqW2V2BIP6cVJ6to5%2FforC3%2Bs7JypTdXZfMm52mq6%2Bnm%2BaabZCmmcrCU7CeoCAouZ2da4av2qfBt5uOu8ZTzqJTw9y69%2Bm0npvsWbK7V6DA1ajLANogHrxUoK1Vn%2FGtv7OkjnfRosyvmNfcbZahtpqg4py2EdifwMKmiqGifcu%2Fo6O6lqfaWY%2B9o6C4gXnLqvYK57aVX5mqm%2BettrKYkbyBl89dmb7oEOGosZatmZ66bqqWy9aULeT2AOptmKFopanbq7LImE25xqHPo5zA5BDVrrGWrZmdvG6Hn8bIpcuqlH29vKCvqVWA2qYQ%2B6OWuISE356h0eSxlaCtVZ7eWbOvpPAEzZzLsFO%2F4LuZorGYozzav7eYoHfFooqNpcziv5WpqVV86KXAr1dzuM72F6mcvpuyoVy7nq7umhD1%2BtDGgZfPXaPM3b%2BZtqlhWuyetMOlkcaBlIqjlMbzrlSgt1WK66i0wJiauItWu7KUy%2B%2B2mJ2smlrdnm20mJoaDp%2FTnqZ937JUnqmestpZv7OlkbiLXYqfmMvgs52fsfjb66KuwVeRxoGD3Kyaz9y6lVyKpKbsmm2UmJoaDp%2FTnlau8K6isLGZm92ebbKcTb3CoC3qn8bcwF),  [People](https://aplicacoes.cidadania.gov.br/vis/data3/v.php?q%5b%5d=oNOclsLerpibuKep3bV%2BgW5g05Kv2rmg2a19ZXR1ZWumaX6JaV2JlmCab2CNrMmim7iareyYsK%2BbjMfDmaWjlMnusm%2BwuqqftHSzr6OgvJxu3bKggOmspKG7qJmpcH2tmo67wKPMo27D3LmnoYOprO6eiImdjsPUmKV4ptLocKKbuJqt7Jh9hWeMiJVjyaCUwdq9lqKDm5vlrLKJq5%2FMxm6lo5TJ7rJvd7uqp5y0brGYoLyBqtKioX27u5Osrait2GmEfpZei5GSzZ6XvOuvmnl4VZvnnW2OpYzHxqbdnGOUq6xlcHiUndqdrL6Zk5SRU96lmMubu6motFWf5ayybpqcuM2Y3aCYhbu7k6ytqK3YaYR%2Bll6LkZLNnpe866%2BaaHheZdyorrqcoLrGW6qrks3gwKebeGxq2JyuspaducdfmmZTwumxVbmDm5vlrLKJnY7D1JileJm%2B58CZd4Oor%2BZcu62nksrUkptxY7ythGabq5ae2KmvtHKTuM2mz3inz%2FCyb3eulqbsnoiJqqLEhK6LfaG867Knr6eYm92YvbCdTYSBW6qrks3gwKebeGxq2JyuspaducdTlV1zy9q9ma%2B7lGqwaax%2Fa122xJTOnKO%2F4W1fXIijmemewMGWXouRkpx0ZbzerpibuJegolrKiZ2Ow9SYpaOUye6yb3eulqbsnoiJqqLE3YTfnqHR5LGVoK1VruitrrpXkbyBo8%2BwpszcwFShtVWg2qYQ%2B6OWuNRTzKKhwuG2l6UL1qzimsBum5x3saXZpKW%2B6K5UfrehrdpZk6%2Bk8ATNnMtghNLcu6ilrJae3lmxs1edvNSm2Z6mfeC6VKKpov0mpbavqk13xqCKsJzR8K734wvYqZmdsm6cpcvTmNeeU83qr6ahwpZa2567s52Wusr2C6%2Bcvu5tmKtohazooL%2BvpI53o6LWsJR9wa6h%2F%2FWho9pcnsOYm8vKl8uhmH3fslSsrait6JrAbpyad8eU1wDgyeSup1xomqeZrLbCrI4aCPYNrFPB4G2kq6qnn%2FOabbCcm7zHnM2m9v7ttpWvaJmpmYm%2FvZ6fuM6Uin%2Biye6uVIKpov0mpbavWn7MwqHeppe%2B37JUoK1Vqt6swL2YoHfGoIqjlMo%2B%2BqClqaha2567s52Wusr2C6%2Bcvu5tmKtohazooL%2BvpI53o6LWsJR9wa6h%2F%2FWho9pZsrtXoMDVqMsA2iAevFSgrVWq6Ju%2Fs7GOg4Gmz6Soy9%2B8VJ1om5visa5um5x3saXZpKW%2B6K5eX5mqm%2BettrKYkbyBl89do8LuwKOdu1Wf5lmzr6TwBM2cy7BTweBtlp2xrZuZq7K8m46Bi1PMoqHC4baXpQvWrOKawG6bnHexpdmkpb7orlR%2Bt6Gt2lmTr6TwBM2cy2CE0ty7qKWslp7eWbGzV5281KbZnqZ94LpUoqmi%2FSaltq%2BqTbrQoIqvmMvfrlSsrada3Jq9t6uOd86Y2LCUyZuul6W1llrdnm27nJbGgabLqfb%2B7bajabX45%2Beiur1hV4GBlc%2BrmMPksJ3%2F6aej2qxtsqZNp9Oi0a%2BUytxtdqu0qJuZf667%2BtrDypTmrW7Z98lmbHpoZ6lsen5ogYeRbZptbY2rp28%3D&ag=m&dt1=2023-02-01) | 03/2023-03/2025 | Download via HTTP | Aggregated families and people | Join the Beneficiary Persons and Beneficiary Families tables in Bolsa Família; create the BENEFICIARIAS_BAIXA_RENDA and BENEFICIARIAS_TOTAL columns for Persons and Families; fill nulls with zero. |
| Bolsa Família value | SAGICAD | 2 | [Link](https://aplicacoes.cidadania.gov.br/vis/data3/v.php?q%5b%5d=r6JtZJCug7BtxKW25rV%2FfmdhhJFkl21kmK19ZnF1ZW2maX7KmZO20qfOnJm%2B6IianbSon7SfrrqqkpKcmcuppsK2iKextVi1mpyuwZxNzsmY2F1zyuDAk522pHa2YH9%2BaV6EkmOXbWSEm8GcobZVetufrL%2BrkbbHlNddmMnuslSqvaGmmZ67sliqkseU1rCYmOGuoK%2BtcHXfmrnBnGiS1KjXYK5%2B3q6noWisot6nbY6kksrAlNiscZqif2Rue2JqrGZ9f15Ny8mY2F1zv%2BGspbCslKDapm2zo6C8gaHfqZ994LuYXcVwoNqlwLNyk7jNps94bsPcuaehg3Ct7qZwyViQuNSYirSbwultdKmtqJnap7yKdFSJkWWbamSNqH1lY2ipot6nbY6Zk7bXn4qin9DgbaKxtKFa3qexb7RovcKf3aJuw9y5p6GDcKDapcCzcmjK1qCNuFTA3MCZXL%2Bdn%2BdZjbucoLbCodl7cIStfWZvdWVtpml%2BdVehv8ahin2Vw9rDoFytoa3eWbvDo5l3xqHOXrCY4a6gr61woNqlwLNyaL3Cn92ibpjuwqFfw1ad2qyybq6VvM9TqqqY0NquoquEcmGraX9%2FZF6HjmObZFPR47KiXHCYm%2ByebcWfksWBc8yjks7vsZOiqaJ4qVnBtpybd9Oi36uXhbuvmpu%2BoXSzp8K7nJ%2FAxGKqn5m87MGYm66Wp6Vrdm6cmcrGU9iyn8mbsqKgcVWf5ayybqWiw81Tz6uXfviImp20qJ%2B0n666qpKSnJnLqabCtoinsbVYtZqcrsGcTc7JmNhdc8rgwJOdtqR4tmB%2FfmlghJFml21khJvBnKG2VWLcmsCzV6S%2FxqGKfamPsYFnenhVruGeu26pnMzPl5J9lcPaw6B2gqOv5p6%2Ft5pcl9dloHFmia12VKG0qJ%2BZp8K6o028z5eTXZjJ7rJUqr2hppmeu7JYqpLHlNawmJjhrqCvrXB135q5wZxoktSo17l5vugQ4aixlq2Ze7K8nJPAxJwt3qXG3MBXgqmi%2FSaltq%2BqTaejeYpllNE%2B9lSLvalpq2l%2Ff2BQncKgLeqfxtzAVIyKe1qhmm2%2BmJ%2FLyqWKoZh9yK6ma3plbKxicKSYmcbTU9yio77uwJWgt1X9GaxttJiaGg6f056mfcuPelxwlq484m2drKGGk2OcblyA0a6gq7pVrN6prsGqjrvQUy3dpn3hrqH%2F9aGj2qxtnnlzd4mUiq2Uz%2B%2B2plysmlrGmr99aV2JlFyNk5TJ6r9UoLdVfN6nsrT62rrKooqq9gbftqNccJauPOJtnayhhpNjnG5cgNGuoKu6VZ7oWY%2BzpZK9JODNpqJ96BDdoLGkWqGabb6Yn8vKpYqhmH3IrqZremVsrGLJvnKp091lmm1miqt%2BYWx5iWqpc31%2BcV2Hu24%3D&ma=mes&ag=m&dt1=2023-03-01) | 03/2023-03/2025 | Download via HTTP | Filtering by state | Filter only the municipalities of "UF"="SP". |
| SISAN | MDS | 3 | [Link](https://www.gov.br/mds/pt-br/caisan/sisan/municipiosaderidosaosisan.pdf) | 2024-2025 | Download via HTTP | PDF to parquet conversion, stacking by day, filtering total rows | Convert .pdf to .parquet; filter only rows with numbers for the "City Code" column and rows with letters for the "State" column; format the "City Code" column to integer type; unite files by day. |
| COMSEA and CAISAN | MDS | 3 | [Link](https://app.powerbi.com/view?r=eyJrIjoiMTc3ZDE3NzMtOWNjZi00ODQ2LWExMTMtMTQ1ZjhiMDkyY2VjIiwidCI6IjNhNzhiMGNkLTdjOGUtNDkyOS04M2Q1LTE5MGE2Y2MwMTM2NSJ9) | 2023 | Webscraping | Stacking and joining indicators, converting html to csv | Convert .html to .csv; create the tables "Caisan_ativos", "Comsea_existe" and "Comsea_ativos"; join the tables; fill nulls with "Not informed". |
| PNAE | FNDE | 3 | [Link](https://www.gov.br/fnde/pt-br/acesso-a-informacao/acoes-e-programas/programas/pnae/consultas/pnae-dados-da-agricultura-familiar) | 2020-2022 | Manual download | Excel to parquet conversion, stacking by year | Union the spreadsheets by year; filter by "UF"="SP and "ESFERA"="MUNICIPAL"; replace the null value in the "UF" column with "SP" for "IBGE"=3518404; clear the values ​​"100% or more" in "Percentage" to 1 and "#VALUE" to '' in "Percentage"; replace null values ​​in "Transferred Value" and "Family Farming Acquisitions Value" with zero; create the columns "30% Transferred Value" (0.30 * TRANSFERRED_VALUE), "Returned Value" ("30% Transferred Value" - "FAMILY_AGRICULTURE_ACQUISITIONS_VALUE") and "Correct Percentage" ("FAMILY_AGRICULTURE_ACQUISITIONS_VALUE" / "TRANSFERRED_VALUE"). |
| EBIA | MDS, IBGE PNADc | 4 | [Link](https://mdsgov-my.sharepoint.com/:x:/g/personal/jose_alexandre_mds_gov_br/EUHTpvVXtARHgXZLMSqFGCIBJ2BZI4ZRb2v6mqtyngcp9g?rtime=3o3M90hX3Ug) | 2009, 2013, 2018, 2023 | Manual download | Column cleaning, year correction | Replace values ​​of rows with year 2008 to 2009; format columns "SGPerc", "IALPerc", "IAMPerc", "IAGPerc" to Brazilian standard ("." para ",") and convert to percentage (divide by 100). |
| Nutritional status | SISVAN | 4 | [Link](https://sisaps.saude.gov.br/sisvan/relatoriopublico/index) | 2020-2025 | Webscraping | Standardization and cleaning, stacking per year | Convert .html to .csv and then to .parquet; create the files for Height x Age and BMI x Age; rename the columns; delete the Totals rows; replace the ‘%’ in the columns with PCT and ‘.’ in the Total (thousands and decimals); insert the columns YEAR, AGE_RANGE, AGE_RANGE_COD; generate the csv file by year; join the Height x Age and BMI x Age files. |
| Food intake | SISVAN | 4 | [Link](https://sisaps.saude.gov.br/sisvan/relatoriopublico/index) | 2015-2024 | Webscraping | Standardization and cleaning, stacking per year | Convert .html to .csv and then to .parquet; create files for the indicators Exclusive Breastfeeding, Continued Breastfeeding, Fruit Consumption, Vegetable Consumption, and Habit of eating meals while watching screens; create the columns "REGION", "STATE_CODE", "STATE", "IBGE_CODE", "MUNICIPALITY", "TOTAL_CASES", "PCT_CASES", "TOTAL_ASSESSED", "YEAR", "AGE_RANGE", "AGE_RANGE_ORDER", "REPORT_TYPE"; merge the files by year. |
| Death declaration | SIM | 5 | [Link](https://opendatasus.saude.gov.br/dataset/sim) | 2015-08/2024 | Manual download | Stacking by year, column standardization, age derivation, state federative unit filtering | The 2015, 2026, 2017, 2023, 2024 databases have the columns in the same order, but the 2018, 2019, 2020, 2021, 2022 databases have the "COUNT" column at the end of the table. Therefore, there are two data sources for each case. And later, the databases are joined with Union; select only the columns ('TIPOBITO', 'DTOBITO', 'HORAOBITO', 'CODMUNNATU', 'DTNASC', 'IDADE', 'SEXO', 'CODMUNRES', 'CODMUNOCOR', 'LINHAII', 'CAUSABAS', 'ATESTADO'); convert AGE to IDADE_ANOS, create the columns UF (from CODMUNRES), ANO_OBITO and MÊS_OBITO (from DTOBITO); filter only rows with UF = '35' (only state of SP); generate the consolidated file with all years. |
| Vaccination Coverage | PNI | 5 | [Link](https://infoms.saude.gov.br/extensions/SEIDIGI_DEMAS_VACINACAO_CALENDARIO_NACIONAL_COBERTURA_RESIDENCIA/SEIDIGI_DEMAS_VACINACAO_CALENDARIO_NACIONAL_COBERTURA_RESIDENCIA.html) | 08/2024 | Manual download | Standardization, cleaning, filtering of columns | Vaccination coverage is calculated by dividing the number of doses administered by the total number of people eligible for vaccination (children under 1 year of age – SINASC database), multiplied by 100.  The municipality of residence, not the location of the vaccination, was used as the assumption.  Fix rows with errors: dTpa Adult -> null; filter State of Residence = "SP" and (Municipality of Residence <> "null", "Totals"); create "Municipality of Residence Code" from "Municipality of Residence"; unpivot columns of vaccine types to lines. |
| Primary Health Care Coverage | SAPS | 5 | [Link](https://egestorab.saude.gov.br/paginas/acessoPublico/relatorios/relHistoricoCoberturaCadastroConsolidado.xhtml) | 01/2021-09/2023 | Manual download | - | Insert date columns (year, month); filter UF = "SP"; create the "APS Coverage Range" column with the ranges: "0 – 35%", "35 – 70%", "70% or more". |
| School Census | INEP | 6 | [Link](https://www.gov.br/inep/pt-br/areas-de-atuacao/pesquisas-estatisticas-e-indicadores/censo-escolar/resultados) | 2021-2024 | Manual download | Stacking by year, column filtering | Union files by year; filter only "UF"="SP" |

^a^ Thematic axes: 1. Demographic; 2. Socioeconomic; 3. FNS policies; 4. Nutritional; 5. Health; 6. Educational.

^b^ The period indicated refers to the time of publication of this article. New updates are being made as announced by the responsible agencies.

IBGE, Brazilian Institute of Geography and Statistics. HDI: human development index. UN, United Nations. RAIS, Annual Report of Social Information. SAGICAD, Secretariat of Assessment, Information Management and Single Registry. MDS, Ministry of Social Development and Assistance, Family and Fight against Hunger. COMSEA, Municipal Food and Nutrition Security Councils. CAISAN, Interministerial Chamber for Food and Nutrition Security. PNAE, National School Feeding Program. FNDE, National Education Development Fund. EBIA, Brazilian Food Insecurity Scale. PNADc, Continuous National Household Sample Survey. SISVAN, Food and Nutrition Surveillance System. BMI, body mass index. SIM, Mortality Information System. PNI, National Immunization Program. SAPS, Secretariat of Primary Health Care. INEP, National Institute of Studies and Educational Research Anísio Teixeira.
